# Unraveling the Function of Lemur Tyrosine Kinase 2 Network

**DOI:** 10.3389/fphar.2019.00024

**Published:** 2019-01-29

**Authors:** Daniel F. Cruz, Carlos M. Farinha, Agnieszka Swiatecka-Urban

**Affiliations:** ^1^Biosystems and Integrative Sciences Institute, Faculty of Sciences, University of Lisboa, Campo Grande, Portugal; ^2^Department of Nephrology, Children’s Hospital of Pittsburgh, Pittsburgh, PA, United States

**Keywords:** LMTK2, PP1, kinase, phosphorylation, signaling pathway

## Abstract

Lemur Tyrosine Kinase 2 (LMTK2) is a recently cloned transmembrane protein, actually a serine/threonine kinase named after the Madagascar primate lemur due to the long intracellular C-terminal tail. LMTK2 is relatively little known, compared to other kinases but its role has been increasingly recognized. Published data show that LMTK2 regulates key cellular events, including endocytic trafficking, nerve growth factor signaling, apoptosis, and Cl^−^ transport. Abnormalities in the expression and function of LMTK2 are associated with human disease, such as neurodegeneration, cancer and infertility. We summarized the current state of knowledge on LMTK2 structure, regulation, interactome, intracellular localization, and tissue expression and point out future research directions to better understand the role of LMTK2.

## Introduction

Protein phosphorylation is an important post-translational modification regulating protein–protein interactions and signal transduction (Manning et al., 2002a; Ubersax and Ferrell, 2007; Ardito et al., 2017). Indeed, the activity of enzymes and receptors is controlled by specific kinases and phosphatases. Kinases are responsible for protein phosphorylation, a reversible process that consists of the addition of a phosphate group PO_4_ from the adenosine 5′-triphosphate (ATP) or guanosine 5′-triphosphate (GTP) to a polar group of various amino acids. This addition modifies the protein from hydrophobic apolar to hydrophilic polar, allowing a conformational change during interaction with other molecules (Ubersax and Ferrell, 2007; Cheng et al., 2011; Ardito et al., 2017). Most protein phosphorylation events occur on hydroxyl groups at the side chain of serine (S), threonine (T), and tyrosine (Y) residues (Ubersax and Ferrell, 2007; Schwartz and Murray, 2011; Ardito et al., 2017). In turn, phosphatases remove the PO_4_ group from phosphoproteins by hydrolyzing phosphoric acid monoesters (Barford, 1996; Zhang, 2002; Ardito et al., 2017).

The human kinome comprises 518 protein kinases and 20 lipid kinases encoded by genes that correspond to 1.7% of the human genome (Manning et al., 2002b; Heath et al., 2003; Duong-Ly and Peterson, 2013). Lemur Tyrosine Kinase 2 (LMTK2) is one of the most recently cloned serine/threonine (S/T) kinases. It is also known as cyclin-dependent kinase-5 (cdk5)/p35 regulated kinase (cprk), kinase/phosphatase/inhibitor-2 (KPI2), brain-enriched kinase (BREK), and apoptosis-associated tyrosine kinase (AATYK)-2. LMTK2 was found almost simultaneously by three different groups. In 2002, Wang and Brautigan aimed to understand the protein complex composed of Inhibitor 2 (Inh2) and Protein Phosphatase 1 (PP1), specifically if Inh2 binds to other partners besides PP1, and identified LMTK2 as one of the interacting proteins by yeast two-hybrid assay (Wang and Brautigan, 2002). In 2003, Kesavapany et al. (2003) found LMTK2 as a binding partner of the cdk5/p35 kinase. In 2004, Kawa et al. (2004) identified LMTK2 as a novel kinase expressed in the brain, using *in silico* search, confirmed by reverse-transcription polymerase chain reaction (RT-PCR) and Northern blot analysis. Here, we present a comprehensive review of published data on LMTK2, identify knowledge gaps, and point out research directions to better understand the role of LMTK2 in physiology and human disease.

## Structure, Specificity, Regulation, and Localization of Lmtk2

**FIGURE 1:**
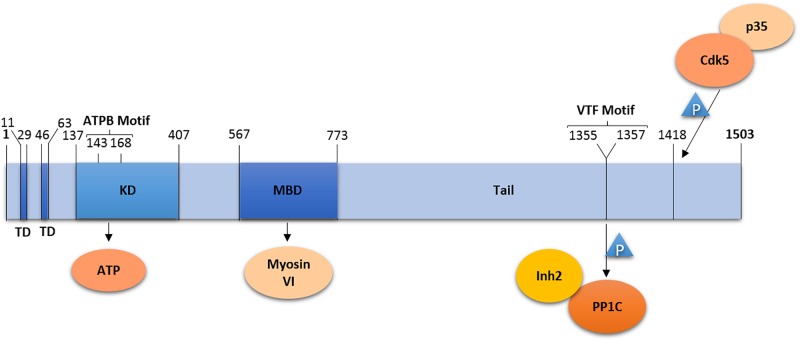
Domain organization of LMTK2. LMTK2 contains two transmembrane domains (TD), followed by a kinase domain (KD) with an ATP binding (ATPB) motif, a Myosin VI binding domain (MBD), and a tail domain. The kinase domain residue K^168^ is critical for LMTK2 catalytic activity. LMTK2 interacts with PP1c via its VTF motif (residues 1355–1357). LMTK2 is phosphorylated in its residue S^1418^, by the complex Cdk5/p35. Numbers indicate amino acid residues.

Lemur Tyrosine Kinase 2 is composed of 1503 amino acid residues forming a short soluble N-terminal domain, followed by two hydrophobic transmembrane helices (residues 11–29 and 46–63), and a kinase domain (residues 137–407) with the ATP binding site (residues 143–168) (Wang and Brautigan, 2002; Nixon et al., 2013) (Figure 1). N- and C-terminal domains as well as the kinase active site are located in the cytosol (Nixon et al., 2013).

Naming of LMTK2 resulted from the sequence homology of the kinase domain with tyrosine kinases. The bioinformatics analysis revealed 60% homology between the kinase domain of LMTK2 and AATYK (Wang and Brautigan, 2002). LMTK2 also shares a putative autophosphorylation site with Src-family kinases, the Y^295^ residue, while the D^265^LALRN motif in LMTK2 is also present in non-Src tyrosine kinases (Kawa et al., 2004). Despite the initial naming, LMTK2 was found to be a serine/threonine kinase (Wang and Brautigan, 2002, 2006). First, phospho-amino acid analysis demonstrated that LMTK2 undergoes auto-phosphorylation on serine and threonine residues, while tyrosine phosphorylation was not observed (Wang and Brautigan, 2002). Second, immunoblotting with anti-phospho-threonine and anti-phospho-serine antibodies showed reactivity with LMTK2 (Wang and Brautigan, 2002). Last, phosphorylation of myelin basic protein (MBP) by LMTK2 was mostly located at serine residues, with a trace at threonine residues; once again, no tyrosine phosphorylation was found (Wang and Brautigan, 2002). Similar results were obtained using a peptide microarray, which demonstrated that LMTK2 interacts with phosphorylated serine and threonine sites in peptides from bovine MBP (Wang and Brautigan, 2006). In fact, the peptide microarray demonstrated that LMTK2 phosphorylates serine and threonine residues preceded or followed by proline (P) residues (Wang and Brautigan, 2006), suggesting similarity with proline-directed kinases. Although these kinases, such as cyclin-dependent kinase (cdk) or glycogen synthase kinase 3 beta (GSK3-β) phosphorylate only those serine/threonine residues that are followed by proline [(S/T)-P-x] (Pelech, 1995; Wang and Brautigan, 2006). LMTK2 also differs from the proline-directed kinases because it is not exclusively specific to proline sites; actually, many of the LMTK2 reactive sites have neighboring basic residues (Wang and Brautigan, 2006).

The C-terminal domain of LMTK2 contains a V^1355^TF motif that binds the catalytic subunit of PP1 (PP1c) necessary for inhibition of PP1 activity (Wang and Brautigan, 2002). The C-terminal domain is also rich in proline residues conforming to seven PxxP-motifs, where x is a variable amino acid (Kawa et al., 2004). The PxxP domains may be involved in regulation of LMTK2 activity, intracellular localization, or substrate recognition, through interaction with SH3 domains of LMTK2-interacting proteins; however, specific SH3 domain containing partners of LMTK2 have not been identified yet.

### Tissue Expression and Subcellular Localization

According to the Human Protein Atlas (HPA) database^1^, LMTK2 is ubiquitously expressed in human tissues (The Human Protein Atlas, 2018a). Northern blot analysis demonstrated high levels of the LMTK2 mRNA in human skeletal muscle while low levels were observed in the brain and pancreas (Wang and Brautigan, 2002). LMTK2 protein was also experimentally detected in human bronchial epithelial (HBE) cells (Luz et al., 2014) and prostate epithelial cells (Shah and Bradbury, 2015b). LMTK2 transcripts were detected in mice, with the most prominent signal found in telenchepalon (Kawa et al., 2004). Indeed, mouse LMTK2 mRNA levels were much higher in the brain than in the skeletal muscle, in contrast to human LMTK2 (Wang and Brautigan, 2002). LMTK2 expression in the mouse brain was detected at all developmental stages, with increased expression during the early postnatal age (weeks 0–2). Similar to human, LMTK2 was found in mouse primary prostate epithelial cells (Shah and Bradbury, 2015b).

Concerning subcellular localization, LMTK2 is enriched on intracellular membranes, especially in organelles involved in the endocytic and recycling pathways (Nixon et al., 2013). Specifically, LMTK2 was found on transferrin-, Rab5-, EEA1-, and Rab11-positive vesicles (Kawa et al., 2004; Chibalina et al., 2007). Confocal imaging and subcellular fractionation studies detected LMTK2 in nuclear and non-nuclear compartments in prostate cancer cells (Shah and Bradbury, 2015b). In differentiated and undifferentiated PC12 cells, derived from pheochromocytoma of the rat adrenal medulla, LMTK2 was enriched at juxta-membranous regions while in differentiated PC12 cells it was also enhanced at the growth cone (Kawa et al., 2004). LMTK2 can also localize at the plasma membrane. It was detected at the apical and basolateral membrane domain in primary differentiated HBE cells derived from cystic fibrosis (CF) lungs and in immortalized CFBE41o- cells, a human CF bronchial epithelial cell line derived from a CF patient homozygous for the F508del-CFTR mutation (Luz et al., 2014). In HeLa cells, LMTK2 was present at the plasma membrane as well as throughout the cytoplasm and it was enriched in cellular extensions and the perinuclear region (Chibalina et al., 2007).

### Regulation of *lmtk2* Gene Expression

The *lmtk2* gene promoter regulates expression of endogenous LMTK2 (Dey and Bradbury, 2017). The sequence GGTGAGTCAGTG upstream of the transcription initiation site of the *lmtk2* gene, conforms to the phorbol 12-myristate 13-acetate (TPA) response element (TRE) (Dey and Bradbury, 2017). Exposure of HEK293, HeLa, and CFBE41o- cells to low concentrations of TPA significantly increased LMTK2 mRNA and protein levels, that were not further increased with higher concentrations of TPA. In fact, exposure to high concentrations of TPA, in CFBE cells, decreased the LMTK2 protein expression below basal levels (Dey and Bradbury, 2017). Phorbol esters regulate gene expression by altering the protein kinase C (PKC) activity. Inhibition of PKC blunted the upregulation of LMTK2 expression by low concentrations of TPA while prolonged exposure of cells to high concentrations of phorbol esters downregulated PKC activity and inhibited LMTK2 expression (Castagna et al., 1982; Sanders and Stern, 1996; Dey and Bradbury, 2017). TPA induces binding of the transcriptional AP-1 complex c-Jun and c-Fos protein families to the TRE in *lmtk2* promoter and these proteins modulate TPA effects on LMTK2 in a PKC dependent manner (Angel and Karin, 1991; Dey and Bradbury, 2017).

### Biosynthetic Processing of LMTK2

Synthesis of membrane proteins occurs on ribosomes associated with the rough ER followed by export in the coat protein complex II (COPII)-coated vesicles. To facilitate this process, LMTK2 contains three arginine residues R^8^RR, located upstream of the first hydrophobic sequence, which functions as a signal peptide targeting LMTK2 to the endoplasmic reticulum (ER) and secretory pathway (Peterson et al., 2003; Kawa et al., 2004; Owji et al., 2018).

Endoplasmic reticulum export is also regulated by specific amino acid residues located in the protein cytoplasmic domain (Butler and Bradbury, 2015). One of the previously identified ER export motifs is characterized by two acidic residues separated by a variable amino acid [(D/E)×(D/E)] (Sevier et al., 2000; Butler and Bradbury, 2015). The di-acidic motif is localized within the cytosolic domain of the protein, facilitating interactions with COPII components (Butler and Bradbury, 2015).This motif, first found in the vesicular stomatitis virus glycoprotein (VSVG) (Sevier et al., 2000), was later demonstrated in the Cystic Fibrosis Transmembrane Conductance Regulator (CFTR) and subsequently in other mammalian proteins (Fu and Sztul, 2003; Wang et al., 2004). Sequence alignment of various mammalian orthologs of LMTK2 revealed presence of a canonical di-acidic motif in the distal portion of the C-terminal tail, with a proximal upstream tyrosine residue, as observed in VSVG sequence in all species (Sevier et al., 2000; Butler and Bradbury, 2015).

Mutations of the di-acidic ER export motif D^1110^-S-E, with D^1110^ and E^1112^ substituted with alanines (A^1110^SA), caused retention of LMTK2 in the ER; however, a small amount of mutant LMTK2 was exported, suggesting presence of another ER export signal (Butler and Bradbury, 2015). The A^1110^SA mutation increased stability of LMTK2 protein compared to the wild type (WT) kinase (half-life ∼3.5 h vs. ∼2.5 h, respectively) (Butler and Bradbury, 2015). Increased half-life of the A^1110^SA mutant suggests that its targeting to the ER-associated protein degradation (ERAD) pathway is reduced, compared to the WT LMTK2. The ER export motif is located downstream of the catalytic and membrane spanning domains, thus proper folding of the mutant LMTK2 may prevent targeting to ERAD, leading to ER accumulation and subsequent increase of the half-life (Butler and Bradbury, 2015).

## The LMTK2 Interactome

Lemur Tyrosine Kinase 2 has been described to interact with different protein partners, as summarized in Table 1. Validation of such protein–protein interactions with endogenously expressed proteins or *in vivo* models confirms the relevance of the LMTK2 interactome in human health and disease.

**Table 1:** LMTK2 interactome.

Interactors	Type of interaction	Function	Cell line	Reference
PP1	Phosphorylation of PP1c-T^320^	Inhibition of PP1c	HeLa, COS7	Manser et al., 2012a,b; Wang and Brautigan, 2002
Inh2	Direct binding (dependent of PP1c)	Regulation of PP1c	HeLa, COS7	Wang and Brautigan, 2002
Cdk5/p35	Phosphorylation of LMTK2-S^1418^	Activation of LMTK2	HeLa, CHO, rat cortical neurons, COS	Kesavapany et al., 2003; Manser et al., 2012b
GSK3-β	Mediated by PP1c	Inhibition of GSK3-β phosphorylation, leading to its activation	HeLa	Manser et al., 2012a
KLC2	Mediated by GSK3-β	Decreases KLC2 phosphorylation and promotes binding to Smad2	HeLa	Manser et al., 2012a
Myosin VI	Direct binding (no phosphorylation)	Regulation of endocytic trafficking pathway	HeLa	Chibalina et al., 2007; Inoue et al., 2008
CFTR	Phosphorylation of CFTR-S^737^	Regulation of CFTR endocytosis	Calu-3, CFBE41o^−^	Luz et al., 2014; Wang and Brautigan, 2006
NGF	Phosphorylation of LMTK2	Down-regulation of LMTK2 activity	PC12	Kawa et al., 2004
BCL2/BCL-xL	Indirect interaction (cell-type dependent)	Regulation of expression of anti- and pro-apoptotic proteins	HME, MCF10A, HCT116, DLD-1	Conti et al., 2017
BIM	Indirect interaction (cell-type dependent)	Regulation of expression of anti- and pro-apoptotic proteins	HME, MCF10A, HCT116, DLD-1	Conti et al., 2017
AR	Direct binding	Inhibition of AR transcriptional activity	LNCaP, Human prostate tissue, HEK293	Shah and Bradbury, 2015b

*PP1, protein phosphatase 1; Inh2, inhibitor 2; Cdk5, cyclin-dependent kinase-5; GSK3-β, glycogen synthase kinase 3 beta; KLC2, kinesin-1 light chain 2; CFTR, cystic fibrosis transmembrane conductance regulator; NGF, nerve growth factor; BCL, B-cell lymphoma; BIM, Bcl-2-interacting mediator of cell death; AR, androgen receptor.*

### PP1, Cdk5/p35, and Cell Signaling

Protein Phosphatase 1 is an ubiquitous serine/threonine phosphatase regulating many distinct processes, including gene transcription, cell cycle progression, protein synthesis, carbohydrate metabolism, neuronal signaling, and muscle contraction (Kelker et al., 2009). The PP1 holoenzyme is composed of PP1c and diverse regulatory subunits that are cell type and subcellular compartment specific (Pelech, 1995). LMTK2 binds directly to PP1c via the C-terminal VTF motif, resulting in an inhibitory phosphorylation of the T^320^ residue in PP1c (Wang and Brautigan, 2002; Manser et al., 2012a,b). LMTK2 interacts with one of the regulatory subunits of PP1c, Inh2 (Wang and Brautigan, 2002; Bollen et al., 2010). The Inh2-LMTK2 interaction is dependent on PP1c, because both PP1 and Inh2 demonstrated default binding in cell extracts (Wang and Brautigan, 2002). In view of this finding, it was suggested that LMTK2 inhibits PP1c activity through phosphorylation of its residue PP1c-T^320^ or through the interaction with Inh2 (Wang and Brautigan, 2002). It was subsequently shown that the LMTK2 kinase activity is required to inactivate PP1c. Studies conducted in Chinese hamster ovary (CHO) cells, HeLa cells and in cultured rat cortical neurons demonstrated that phosphorylation of LMTK2 on the S^1418^ residue, in mouse sequence, activates the kinase (Manser et al., 2012b). In human sequence, this residue corresponds to S^1450^, however, we will always mention as LMTK2-Ser^1418^ to remain consistent with the literature and to a better understanding. A bioinformatic analysis, using NetPhosK database, predicted that cdk5/p35 phosphorylates S^1418^ with the highest score. This prediction was corroborated with the observation that the LMTK2-S^1418^ precedes a proline and thus is phosphorylated by proline-directed kinases, such as the cdk5/p35 complex. Bioinformatics analysis also predicted that GSK3-β and p38 phosphorylate LMTK2-S^1418^, although these predictions have not been experimentally validated (Manser et al., 2012b). *In vitro* studies confirmed that modulation of the cdk5/p35 activity alters LMTK2-S^1418^ phosphorylation. Studies in HeLa cells, where cdk5 is present in an inactive form due to absence of p35, demonstrated that transfection of p35 increased LMTK2-S^1418^ phosphorylation. However, LMTK2-S^1418^ was also phosphorylated at low levels in the absence of the cdk5/p35 complex, suggesting that other kinases may also phosphorylate LMTK2-S^1418^ in non-neuronal cells (Manser et al., 2012b). Although the LMTK2-S^1418^ is located only 91 amino acid (90 aminoacids in human sequence) residues apart from the PP1c-binding motif, mutation of this residue only affects the phosphorylation of PP1c-T^320^ and does not prevent the LMTK2-PP1c interaction (Manser et al., 2012b). Based on the data, a model has been proposed where cdk5/p35 activates LMTK2 by phosphorylating the S^1418^ residue that in turn stimulates LMTK2 binding and inhibition of PP1 by phosphorylation of the PP1c-T^320^ (Manser et al., 2012b).

### Kinesin 1 and the TGF-β Signaling Pathway

Lemur Tyrosine Kinase 2 controls the kinesin-1 mediated transport along microtubule filaments, including the canonical TGF-β mediator, Smad2. Kinesin-1 is a heterotetramer composed of two motor subunits also known as kinesin-1 heavy chains (KHC) and two associated kinesin-1 light chains (KLC) (Rahman et al., 1998; DeBoer et al., 2008). GSK3-β phosphorylates one of the KLC, KLC2, leading to release of the protein cargo and inhibition of kinesin-1 mediated transport (Morfini et al., 2002). The signaling pathway that controls GSK3-β phosphorylation of KLC2 involves cdk5/p35-mediated inhibitory phosphorylation of PP1c-T^320^, which in turn induces inhibitory phosphorylation of GSK3-β-S^9^. Several lines of evidence support the role of LMTK2 in this pathway. First, LMTK2 contains the PP1c-binding motif. Second, transfection of LMTK2 indirectly increased GSK3-β-S^9^ phosphorylation by mediating the inhibitory phosphorylation of PP1c-T^320^. Third, transfection of LMTK2 decreased KLC2 phosphorylation and promoted binding of a known KLC2 cargo, Smad2, necessary for activation of the canonical TGF-β signaling pathway (Manser et al., 2012a).

### Myosin VI and Endocytic Trafficking

Lemur Tyrosine Kinase 2 interacts with myosin VI, the minus-end directed actin-associated motor protein critical for trafficking in the endocytic and secretory pathway (Chibalina et al., 2007; Inoue et al., 2008). LMTK2 is the first transmembrane protein and kinase identified to directly bind to myosin VI. The myosin VI binding site in LMTK2 is located in a region downstream of the kinase domain (residues 567–773), first discovered by an yeast two-hybrid screen and later confirmed in cultured cells (Chibalina et al., 2007; Inoue et al., 2008). The LMTK2 role in endocytic trafficking does not involve phosphorylation of myosin VI (Tumbarello et al., 2013).

Lemur Tyrosine Kinase 2 binds to the WWY motif in the C-terminal tail of myosin VI and shares the binding site with the multifunctional adaptor protein Disabled-2 (Dab2) (Chibalina et al., 2007; Inoue et al., 2008). Myosin VI recruits Dab2 and LMTK2 to different vesicular compartments. Recruitment of myosin VI to the clathrin-coated structures requires Dab2 while LMTK2 recruitment is necessary for transport from early endosomes to the recycling compartment (Morris et al., 2002; Chibalina et al., 2007; Spudich et al., 2007). LMTK2 is present on endocytic and recycling vesicles, namely a subset of early endosomes, where it colocalizes with Rab5, early endosomal antigen 1 (EEA1) and Rab11, while myosin VI is restricted to Rab5-positive early endosomes (Hasson, 2003; Chibalina et al., 2007). Knockdown of LMTK2 resulted in enlargement, swelling, ring-like appearance, and altered function of vesicles involved in the endocytic trafficking pathways in HeLa cells (Chibalina et al., 2007). Moreover, LMTK2 binding to myosin VI was required for endocytic trafficking of the transferrin receptor (Inoue et al., 2008). Loss of LMTK2 in the appropriate vesicular regions, induced by the ER export motif mutation A^1110^SA, affected membrane trafficking, characterized by reduced recycling of transferrin from the cells without affecting its endocytosis (Butler and Bradbury, 2015). The impact of A^1110^SA on transferrin trafficking was similar to the results obtained after LMTK2 knockdown (Chibalina et al., 2007).

### CFTR and Cl^−^ Transport

Lemur Tyrosine Kinase 2 interacts with CFTR and mediates its inhibitory phosphorylation and endocytosis, and inhibits the CFTR-mediated Cl^−^ transport. CFTR is a member of the ATP binding cassette (ABC) transporter family that functions as a cAMP-activated Cl^−^ ion channel (Riordan et al., 1989; Gregory et al., 1990). CFTR was identified as an interacting partner of LMTK2 through a microarray where the peptide containing the S^737^ residue, corresponding to a sequence in the regulatory domain of CFTR, showed the strongest reactivity with LMTK2 (Wang and Brautigan, 2006).

The interaction between CFTR and LMTK2 was predicted to occur at the plasma membrane (Wang and Brautigan, 2006). The prediction was later experimentally validated in human airway epithelial Calu-3 cells where the endogenous LMTK2 co-immunoprecipitated with endogenous CFTR specifically at the apical plasma membrane (Luz et al., 2014). Additionally, depletion of LMTK2 increased the steady-state plasma membrane abundance and activity of CFTR in the primary HBE cells. Increased CFTR expression at the plasma membrane, after LMTK2 knockdown resulted from decreased CFTR endocytosis, suggesting that endogenous LMTK2 decreases the CFTR-mediated Cl^−^ secretion, at least in part by decreasing density of the CFTR Cl^−^ channels at the cell surface. This is the first evidence that CFTR endocytosis can be regulated, explaining the mechanism of phospho-dependent inhibitory effect of CFTR-S^737^ on the CFTR mediated Cl^−^ secretion (Wilkinson et al., 1997). LMTK2 depletion increased the efficacy of the CFTR corrector VX-809 (Lumafactor), currently a component of Orkambi, a FDA approved drug for CF patients homozygous for the most common disease-causing mutation F508del (Luz et al., 2014). These data suggest that interfering with the LMTK2 mediated inhibitory phosphorylation of CFTR may increase efficacy of CF-directed therapy.

### The Nerve Growth Factor (NGF) and Neuronal Differentiation

Lemur Tyrosine Kinase 2 interaction with NGF inhibits neuronal differentiation. The specific distribution and phosphorylation of LMTK2 in the brain tissue first suggested that it may regulate early postnatal brain function (Kawa et al., 2004). Endogenous LMTK2 is phosphorylated upon stimulation with NGF in PC12 cells, possibly by a PKC-dependent mechanism, although a direct interaction between LMTK2 and PKC has not been demonstrated (Kawa et al., 2004). The NGF-stimulated phosphorylation down-regulates LMTK2 activity, resulting in activation of extracellular-signal-regulated kinases (ERK), leading to neurite outgrowth (Kawa et al., 2004).

### The B-Cell Lymphoma (BCL) Proteins and Apoptosis

It has been suggested that LMTK2 inhibits cytotoxicity by interacting with the apoptotic and survival pathways. A high-throughput siRNA-based screen recently identified LMTK2 as a novel anti-apoptotic sensor (Conti et al., 2017). LMTK2 knockdown reduced the expression of anti-apoptotic B-cell lymphoma-2 (BCL2) and B-cell lymphoma-extra-large (BCL-xL) proteins and increased the expression of pro-apoptotic BCL2-interacting mediator of cell death (BIM) protein. While LMTK2-dependent regulation of BIM was more evident in non-cancer cell lines, other members of the BCL2 family, such as BCL2 and BCL-xL, were regulated by LMTK2 mainly in fully transformed cancer cells. Furthermore, LMTK2 knockdown enhanced the cytotoxic effect of apoptosis inducing ligands, such as the tumor necrosis factor-related apoptosis-inducing ligand (TRAIL) and etoposide, among others (Conti et al., 2017).

### The Androgen Receptor (AR) and Prostate Development

Lemur Tyrosine Kinase 2 interacts with AR and inhibits its transcriptional activity (Shah and Bradbury, 2015a,b). AR promotes cell growth and plays an important role in prostate development (Chang et al., 1988; Mangelsdorf et al., 1995). The LMTK2-AR interaction was demonstrated by co-immunoprecipitation and Proximity Ligation Assay (PLA) in LNCaP cells, an androgen-sensitive human prostate adenocarcinoma cell line. Localization of the complex was dependent on the androgen ligands: under androgen deprivation, it was predominantly extra-nuclear and in presence of androgens the complex translocated to the nucleus. The LMTK2-AR complex was also detected in human prostate tissue (Shah and Bradbury, 2015b). Inhibition of the AR transcriptional activity by LMTK2 was demonstrated by luciferase reporter assay in HEK293 cells (Shah and Bradbury, 2015b).

## Role of LMTK2 in Human Disease: Potential Biomarker and Therapeutic Target

The role of LMTK2 in cell signaling, endocytic trafficking, apoptosis, and Cl^−^ transport explains why its dysregulation is associated with neurodegeneration, cancer, and infertility. Below, we discuss the role of LMTK2 in human disease and its potential to become a biomarker and a therapeutic target.

### Prostate Cancer

The role of LMTK2 in prostate cancer is well established (Eeles et al., 2008; Guy et al., 2009; Waters et al., 2009; Harries et al., 2010; Wang et al., 2013; Shui et al., 2014; Shah and Bradbury, 2015a,b; Hao et al., 2016; Jiang et al., 2016). The *lmtk2* gene was identified as one of seven *loci* associated with prostate cancer in a genome-wide association study (GWAS) using blood DNA from 1854 individuals with prostate cancer detected before 60 years of age or with a family history of the disease (Eeles et al., 2008; Guy et al., 2009). The LMTK2 expression had no significant ethnic heterogeneity, as demonstrated in multi-ethnic prostate cancer populations from California with African and Latino background, or from Hawaii from native, Japanese or European descent (Waters et al., 2009). Furthermore, the LMTK2 single nucleotide polymorphism (SNP), rs6465657 was inversely associated with the risk of non-fatal prostate cancer and the cancer-specific mortality (Shui et al., 2014).

The expression of *lmtk2* gene was studied in benign prostate hyperplasia (BPH) and prostate cancer cells by RT-PCR. The *lmtk2* gene expression was decreased by 68% in tissue with prostate adenocarcinoma, when compared to BPH (Harries et al., 2010). In addition, LMTK2 protein abundance was undetectable or low in prostate cancer tissues, compared to very high expression in non-malignant tissue (Shah and Bradbury, 2015b). Additionally, a direct association between LMTK2 and tumor forming capacity was assessed in LNCaP cells. LMTK2 knockdown demonstrated significantly higher colony-forming capacity and increased cell viability, suggesting that LMTK2 plays a role in the regulation of cell proliferation (Shah and Bradbury, 2015b). This idea is corroborated by the fact that LMTK2 regulates the expression of several proteins involved in apoptosis, as described above.

In view of the above data, LMTK2 has a potential to serve as a novel biomarker in prostate cancer that, in contrast to the prostate specific antigen (PSA), can distinguish between BPH and prostate cancer. Furthermore, targeted activation of LMTK2 in the prostate tissues by small molecules could decrease the AR-proliferative activity, preventing the prostate cancer cell growth.

### Other Cancers

The role of LMTK2 in apoptosis suggests that its dysregulation may be involved in other forms of cancer as well. In fact, LMTK2 mRNA is expressed in all cancer tissues according to The Cancer Genome Atlas (TCGA) (The Human Protein Atlas, 2018b). The LMTK2 protein levels differ in a variety of cancer cells. Levels are high in colorectal and ovarian cancer while they are low in lymphoma, lung, testis and renal cancer (The Human Protein Atlas, 2018b). Thus, LMTK2 has a potential to serve as a prognostic biomarker (The Human Protein Atlas, 2018b). For example, in renal cancer, LMTK2 indicates favorable prognosis because higher expression correlated with higher survival probability. By contrast, higher LMTK2 levels were associated with reduced survival in ovarian cancer (The Human Protein Atlas, 2018b).

Whole-exome sequencing (WES) followed by the Protein Variation Effect Analyzer (PROVEAN) modeling in pulmonary sarcomatoid carcinoma (PSC) cells identified deleterious effects of mutations in the *lmtk2* gene in 2 of 10 patients (Liu et al., 2016). PSC is an aggressive and poorly differentiated, non-small-cell lung carcinoma responsible for 0.1–0.4% of all lung cancer cases (Brambilla et al., 2001; Liu et al., 2016; Karim et al., 2018). A mutation in the *lmtk2* gene was also found in patients with lung adenocarcinoma (Seo et al., 2012). Future studies are needed to experimentally validate the diagnostic and prognostic potential of LMTK2 in different forms of cancer.

### Male Infertility and Contraception

Animal studies suggest a strong association between LMTK2 and male fertility (Kawa et al., 2006; Sakugawa et al., 2009). Studies conducted with LMTK2 knockout (KO) mice demonstrated that LMTK2 plays an essential role in spermatogenesis. Although the KO animals had normal phenotype at birth and experienced normal growth, they had azoospermia (Kawa et al., 2006). Normal number and morphology of testicular somatic cells and intact hormonal levels in the LMTK2 KO mice suggest that impaired spermatogenesis was directly caused by a defect in germ cells. Indeed, it was observed that the first step of spermatogenesis – the differentiation of spermatogonia to round spermatids – occurred normally in these animals; however, further differentiation of spermatids was largely suppressed (Kawa et al., 2006). Thus, it has been hypothesized that LMTK2 is necessary for the morphological progression of postmeiotic germ cells, a process that includes mitochondrial compaction and expulsion of the cytoplasm. This hypothesis was experimentally validated by examination of the wild-type mice, in which LMTK2 expression was higher between 2 and 3 weeks after birth, the time of the late phase spermatogenesis, possibly after the generation of round spermatids (Kawa et al., 2006). Additionally, the *lmtk2* gene was predicted as a target for heat-sensitive micro (mi)RNAs in pachytene spermatocytes (Yadav et al., 2018). However, a study that examined the association of LMTK2 and infertility in humans demonstrated that the nine SNPs in exon 11 of the *lmtk2* gene identified in 18 Japanese men with azoospermia were not causative (Sakugawa et al., 2009).

Lemur Tyrosine Kinase 2 is a known inhibitor of PP1 that regulates sperm motility (Chakrabarti et al., 2007; Fardilha et al., 2011). Hence, LMTK2 emerges as a potential target for both reproductive contraceptives, such as an inhibitor of sperm motility, and infertility treatment in men, by regulating the activity of PP1. However, more studies are needed to better understand how LMTK2 regulates spermatogenesis.

### Cystic Fibrosis

Cystic fibrosis (CF) is the most common lethal autosomal recessive disease in Caucasians (Grody and Desnick, 2001). The most common disease-associated mutation in the *cftr* gene F508del, is caused by the in-frame deletion of three nucleotides encoding phenylalanine at position 508. This mutation, present in 90% of CF patients in at least one allele, leads to an intracellular processing defect and retention of CFTR in the ER. The corrector VX-809 (part of the FDA-approved drug Orkambi) partially rescues the processing defect of F508del-CFTR and allows maturation while passing through the Golgi complex and trafficking to the cell membrane in cultured cells (Van Goor et al., 2011, 2014; Clancy et al., 2012; Ren et al., 2013; Wainwright et al., 2015). F508del-CFTR forms a Cl^−^ channel regulated by cAMP but has dramatically different gating properties compared to the wild-type CFTR (Dalemans et al., 1991; Swiatecka-Urban et al., 2005). The FDA-approved drug Kalydeco^TM^ (Ivacaftor; VX-770) potentiated membrane-resident F508del-CFTR channel “open” probability (Accurso et al., 2014). Clinical trials show that the combined use of corrector VX-809 and potentiator VX-770 (Orkambi) improved the percent of predicted forced expiratory volume in 1 second (FEV1), decreased the rate of pulmonary exacerbations, and reduced the rate of events leading to hospitalization or use of intravenous antibiotics in patients with two F508del copies (Wainwright et al., 2015). Clinical trial evaluating a different corrector VX-661 (Tezacaftor) with VX-770 in patients with two F508del copies showed results similar to Orkambi [Donaldson et al., 2013; Two Phase 3 Studies of the Tezacaftor/Ivacaftor Combination Treatment Met Primary Endpoints with Statistically Significant Improvements in Lung Function (FEV1) in People with Cystic Fibrosis, 2017] – with this combination also securing FDA approval under the brand name Symdeko. Although this is seen as a very important step forward, improved treatment efficacy is still needed for these patients.

While at the cell membrane, the corrector-rescued F508del-CFTR has reduced membrane residence and current therapies do not improve this defect (Swiatecka-Urban et al., 2005). LMTK2 depletion facilitates the VX-809 mediated trafficking of F508del-CFTR to the apical plasma membrane and rescue of the CFTR-mediated Cl^−^ secretion across human bronchial epithelial cells (Luz et al., 2014). Since LMTK2 facilitates CFTR endocytosis, these effects could result from decreased CFTR endocytosis after LMTK2 depletion. Thus, targeting the LMTK2-mediated inhibitory phosphorylation of CFTR-S^737^ could serve as a novel approach to study the plasma membrane stability defect of F508del-CFTR. It could also help to design new pharmacological approaches to stabilize rescued F508del-CFTR and improve the efficacy of the corrector/potentiator strategy (Luz et al., 2014).

### Neurodegeneration

Neurodegeneration represents an irreversible structural and functional damage of neurons that can lead to cell death. It is the hallmark of several central nervous system disorders, including Alzheimer’s and Parkinson’s diseases (Przedborski et al., 2003). Bencze et al. (2018) recently reviewed the role of LMTK2 in neurodegeneration. The authors proposed three LMTK2-dependent mechanisms of neurodegeneration: (i) disruption of axonal transport, mediated by aberrant LMTK2-kinesin-1 interaction; (ii) hyperphosphorylation of Tau protein by LMTK2, mediated by cdk5 and GSK3-β; and (iii) regulation of apoptosis by LMTK2 (Bencze et al., 2018). The authors proposed that modulation of LMTK2 expression could be considered as a promising novel therapeutic target for neurodegenerative states, such as Alzheimer disease (Bencze et al., 2018).

## Conclusion

Although LMTK2 is still incompletely characterized, its role in key biological functions has been well established by strong scientific evidence. LMTK2 works as a multifunctional adaptor, being involved on very distinct signaling pathways and cell mechanisms, such as endocytosis, apoptosis, channel trafficking and cell differentiation. Improved characterization of LMTK2 holds the potential to enhance our knowledge about the molecular basis of human disease and discover novel diagnostic and prognostic biomarkers. Targeting the LMTK2 interactome could also advance treatment for cancer, neurodegeneration, infertility, and cystic fibrosis and create novel strategies for contraception.

## Author Contributions

DC was involved in the planning and writing of the review manuscript. CF and AS-U were involved in the planning, writing and review process.

## Conflict of Interest Statement

The authors declare that the research was conducted in the absence of any commercial or financial relationships that could be construed as a potential conflict of interest.

## Footnotes

1 https://www.proteinatlas.org/
